# Do changes in social and economic factors lead to changes in drinking behavior in young adults? Findings from three waves of a population based panel study

**DOI:** 10.1186/1471-2458-14-928

**Published:** 2014-09-08

**Authors:** Frederieke S van der Deen, Kristie N Carter, Sarah K McKenzie, Tony Blakely

**Affiliations:** Department of Public Health, University of Otago, PO Box 7343, Wellington, New Zealand; Social Psychiatry & Population Mental Health Research Unit, University of Otago, PO Box 7343, Wellington, New Zealand

**Keywords:** Alcohol, Young adulthood, Social, Economic, Transition, Longitudinal

## Abstract

**Background:**

Social and economic measures in early childhood or adolescence appear to be associated with drinking behavior in young adulthood. Yet, there has been little investigation to what extent drinking behavior of young adults changes *within young adulthood* when they experience changes in social and economic measures in this significant period of their life.

**Methods:**

The impact of changes in living arrangement, education/employment, income, and deprivation on changes in average weekly alcohol units of consumption and frequency of hazardous drinking sessions per month in young adults was investigated. In total, 1,260 respondents of the New Zealand longitudinal Survey of Family, Income and Employment (SoFIE) aged 18-24 years at baseline were included.

**Results:**

Young adults who moved from a family household into a single household experienced an increase of 2.32 (95% CI 1.02 to 3.63) standard drinks per week, whereas those young adults who became parents experienced a reduction in both average weekly units of alcohol (β = -3.84, 95% CI -5.44 to -2.23) and in the frequency of hazardous drinking sessions per month (β = -1.17, 95% CI -1.76 to -0.57). A one unit increase in individual deprivation in young adulthood was associated with a 0.48 (95% CI 0.10 to 0.86) unit increase in average alcohol consumption and a modest increase in the frequency of hazardous drinking sessions (β = 0.25, 95% CI 0.11 to 0.39).

**Conclusions:**

This analysis suggests that changes in living arrangement and individual deprivation are associated with changes in young adult’s drinking behaviors. Alcohol harm-minimization interventions therefore need to take into account the social and economic context of young people’s lives to be effective.

## Background

International research has linked young adulthood with a peak in the consumption of alcohol, heavy drinking sessions, and frequent drunkenness [1–4]. These hazardous drinking patterns among youth seem to be increasing in many countries around the world [5–7]. Young adulthood is also associated with changes in social and economic roles and responsibilities. It is a stage in life where many have left or are about to leave high school and build further on their education, start a career, move out of the family home, experience increased financial responsibilities, and/or start their own family. Understanding how, and especially, to what extent these *changes* are associated with *changes* in drinking behavior *within young adulthood* may identify entry points for harm-minimization interventions and social policy.

There is a growing body of longitudinal research on the association between social and economic predictors in early childhood or adolescence with hazardous alcohol use outcomes in young adulthood [8–13]. The results from these studies are generally mixed, with some studies suggesting a relation between high income and/or education with higher levels of drinking in young adulthood, and other studies suggesting an association between low income or school leaving with hazardous drinking outcomes in young adulthood. A number of studies also found strong associations between hazardous alcohol use in young adulthood and social factors such as living situation and family relations [9–12].

However, there exists a gap in this literature. Although some studies examined trajectories over time in either exposure measures or outcome measures, most longitudinal studies have not investigated the impact of *changes* in both social and economic exposure measures and *changes* in drinking behaviors *within young adulthood* using methods that control for time-invariant unobserved confounders [14–16]. Using repeated measures data from the same individuals over time that captures change in both the exposure and outcome provides stronger information for causal inference than cross-sectional studies or cohort studies where individuals do not change their exposure to social and economic factors [16].

The few studies that have investigated the association between changes in social factors within young adulthood with changes in young adult’s drinking behaviors have shown that moving out of the family home and moving in with a spouse [17–19] or becoming a parent [20–22] is associated with a decrease in alcohol consumption and frequency of hazardous drinking sessions. The opposite pattern is found for young adults who move out of the family home into other types of living arrangements (e.g. living with an unmarried partner, by themselves or with flatmates) [17, 19].

In this study, based in New Zealand where highest hazardous drinking rates are observed in young adulthood [7], we estimate the impact of changes in both social and economic factors (living arrangement, education/employment status, income, and deprivation) on two different measures of alcohol use (average weekly alcohol consumption and frequency of hazardous drinking sessions) in young New Zealand adults aged 18 to 24 years old at baseline. We used three years of data from the longitudinal Survey of Family, Income and Employment (SoFIE).

## Methods

### Data

The present study is a longitudinal analysis of wave 3 (2004/05), 5 (2006/07) and 7 (2008/09) from SoFIE. In short, SoFIE is a nationally representative longitudinal survey of the usually resident population living in private dwellings in New Zealand and was conducted between 2002 and 2010 [23]. In annual face-to-face interviews, information on individual and family factors such as household composition, income, deprivation, education, and employment status was obtained. In waves 3, 5 and 7 of SoFIE, a detailed health module included questions on alcohol consumption behavior. The initial SoFIE sample comprised approximately 11,500 responding private households (response rate of 77%) with 22,000 adults responding in wave 1 and just over 16,000 in wave 7. The present analysis was restricted to respondents aged 18-24 years old at wave 3 (aged 20-26 at wave 5 and 22-28 at wave 7) who responded in at least two of the three waves (N = 1260).

### Social and economic exposure measures

#### Living arrangement

A young adults living arrangement was classified at each wave as living in a family household in a child role (*reference category*) [1], living in a family household in a parent role (i.e. becoming parent) [2], living in a couple (only or also with others) [3], living in a one person household [4], or living in another multiperson household (with non-family members – flatmates, friends) [5].

#### Education and employment status

A respondent’s education and employment status was classified as follows: enrolled in education (secondary school, vocational training or university) and not employed (*reference category*) [1], enrolled in education and employed [2], not in employment nor educational training (NEET) [3], or employed, and not in education [4]. Employment included both part and full time work. Education and employment were combined into one exposure variable in this study because many young people who are enrolled in education might also have a part time job and those who are employed may also be participating in further study.

#### Income

Annual personal income was derived by combining gross annual incomes from employment earnings, self-employment earnings, government transfers, interest from bank and/or other accounts, personal investments, private superannuation and pension schemes, and other regular or irregular received income sources. Personal income was adjusted for inflation and log-transformed prior to modelling.

#### Area deprivation

Area deprivation was measured using NZDep 2001, a census based measure of area deprivation in New Zealand which uses an index of nine census variables (e.g. benefit receipt, income) averaged across about 100 people living in the smallest census enumeration area (i.e. meshblock), and classified into deciles [24]. Each respondent’s address was assigned to an NZDep score (this was allowed to change if respondents moved between waves), modelled as continuous deciles for fixed effects regressions and classified into quintiles for descriptive analyses (quintile 5 indicating the most deprived category).

#### Individual deprivation

Individual level of deprivation was measured using the New Zealand Individual Deprivation Index (NZiDep), which is a composite score from eight items such as not being able to afford fruit and vegetables or using a food bank [25]. It was asked of all respondents in waves 3, 5 and 7, and was scored as zero (no deprivation) up to eight (reporting ‘yes’ to all measures of deprivation). This was categorised as one, two, three to four, or five or more deprivation factors for descriptive purposes. In the longitudinal fixed effects regression analysis, this was modelled as a continuous variable (0 to 8).

### Outcome measures

#### Average weekly alcohol consumption

As part of the SoFIE health module in waves 3, 5 and 7, all young adults were asked if they had consumed an alcoholic drink in the past 12 months. If they answered ‘yes’ respondents were asked how many days in the last four weeks they had consumed alcohol and how many standard drinks they consumed on a typical day. A standard drink was defined using standard definitions, as a can or small bottle of beer, a small glass of wine or a single nip of spirits [26]. This information was used to calculate the average weekly consumption of alcohol.

#### Frequency of hazardous drinking

It has been suggested that frequency of hazardous alcohol consumption sessions or drunkenness is the best indicator of hazardous drinking behavior in young adults [27]. Respondents who had consumed alcohol in the last 12 months were asked if they ever had eight or more (for males) or six or more (for females) standard drinks per occasion in the last month. This was defined as hazardous drinking. For descriptive purposes, the frequency of hazardous drinking per month (for drinkers only) was categorised as: never hazardous [1], hazardous drinking monthly [2], hazardous drinking twice per month [3], hazardous drinking weekly [4] or hazardous drinking (almost) daily [5]. The frequency (number of occasions) of hazardous drinking episodes per month (0 to 28 days) was treated as a continuous variable in the fixed effects regression modelling. Note that the definition of hazardous drinking (at the time when the data was collected) deviates from the current drinking advice of not more than four standard drinks in one drinking session for women and no more than five for men [26]. The number of hazardous drinkers in this sample may therefore be underestimated.

### Analyses

All analyses were conducted using individual unit data (SoFIE data Wave 1 to 8, version 1) in SAS Enterprise Guide 4.3 in the Statistics New Zealand data laboratory, Wellington. Fixed effects models, applied to longitudinal data, control for all time-invariant confounding, both measured and unmeasured, by using only the changes in exposure occurring within individuals to estimate the outcome. Therefore, effect estimates are not biased by unmeasured heterogeneity (between individuals) [14–16]. In the present study, fixed effects regression models were used to estimate the relationship between a change over time in each of the social and economic exposure variables (living arrangement, education/employment status, income, and deprivation) and a change in the two drinking behavior outcome variables (average weekly alcohol consumption and frequency of hazardous drinking sessions) in young adult SoFIE respondents (aged 18-24 at baseline (wave 3)). The final model for each of the outcome variables was fully adjusted for all of the social and economic exposure variables.

## Results

In a previous descriptive report, we have shown higher levels of drinking in 15-24 year old males and females who did not have a school qualification, or lived in a flatting situation or in a one person household, whereas young respondents who were inactive in the labour force reported lower levels of alcohol consumption (Carter, Filoche & McKenzie: *Alcohol and Young People: A Descriptive Analysis of Changes in Alcohol use in Young New Zealanders from the Statistics New Zealand Longitudinal Survey of Family Income and Employment (SoFIE) and the SoFIE-Health sub-Study,* forthcoming). The distribution of drinking among 18-24 year old respondents at baseline (wave 3) and at wave 5 (aged 20-26) and wave 7 (aged 22-28), and transitions between waves, is shown in Table 1.

**Table 1:** **Distribution and transitions in average weekly consumption and frequency in hazardous drinking for all young adult respondents (N = 1,260; percentages in parentheses for non-missing respondents and data)**

Drinking behavior variable/transition ^a,b^	Wave 3	Wave 3 to 5 transition	Wave 5	Wave 5 to 7 transition	Wave 7
**Average weekly consumption**	**N = 1,050 (current drinkers only)**	**N = 1,130 (drinkers in both waves)**	**N = 1,005 (current drinkers only)**	**N = 1,140 (drinkers in both waves)**	**N = 1,000 (current drinkers only)**
Mean (standard deviation)	7.45 (11.48)		7.10 (11.08)		7.27 (11.56)
Increase by > 2 drinks		520 (27.4%)		265 (23.2%)	
Increase by 1-2 drinks		210 (18.6%)		170 (14.9%)	
No change		195 (17.3%)		185 (16.2%)	
Decrease by 1-2 drinks		160 (14.2%)		210 (18.4%)	
Decrease by > 2 drinks		255 (22.6%)		310 (27.2%)	
Missing data ≥ 1 wave		135		125	
**Frequency hazardous drinking**	**N = 1,055 current drinkers**	**N = 950 drinkers in both waves**	**N = 1,065 current drinkers**	**N = 955 drinkers in both waves**	**N = 1,050 current drinkers**
Mean (standard deviation)	1.86 (2.96)		1.89 (3.10)		1.77 (2.99)
Never	515 (48.8%)		525 (49.3%)		530 (50.5%)
Monthly	160 (15.2%)		155 (14.6%)		165 (15.7%)
Twice per month	105 (10.0%)		105 (9.9%)		100 (9.5%)
Weekly	165 (15.6%)		170 (16.0%)		150 (14.3%)
Almost daily to daily	110 (10.4%)		110 (10.3%)		105 (10.0%)
More frequent hazardous drinking		250 (26.3%)		265 (27.7%)	
No change		435 (45.8%)		445 (46.6%)	
Less frequent hazardous drinking		265 (27.9%)		245 (25.7%)	
Missing data ≥ 1 wave		320		310	

^a^Note that all counts are random rounded to a near multiple of 5 as per Statistics New Zealand requirements; therefore numbers in each column do not sum exactly to N = 1,260.

^b^Note that N is different for average alcohol consumption and frequency of hazardous drinking, as the frequency in hazardous drinking was measured in current drinkers only.

The prevalence of drinking (having consumed alcohol in the past 12 months) at wave 3 was 87.5%, and this remained stable over time. Very few (between 4 - 5%) young adults stopped or started drinking between waves. The mean overall average weekly alcohol consumption among young adults was stable at 7 to 8 units a week across waves. The large standard deviations reflect the wide and skewed distribution.

Approximately 50% of the young adult drinkers reported hazardous drinking sessions at least once per month. Over half of the 18-24 year old respondents at wave 3 changed the frequency of hazardous drinking between waves, but these were equally split between moving into more or less frequent hazardous drinking. The mean number of hazardous drinking episodes per month was stable at 1.9 to 1.8 per month across waves 3, 5 and 7.

Table 2 shows the distribution and changes over time of the exposures. Slightly more than a third of the young respondents experienced change in their living arrangement status between waves. Approximately between 42-47% of the young adults changed their level of employment and/or education between waves. There was an increase in mean personal income over the study period. Around 40% of respondents experienced either a decrease or increase in log personal income greater than half a standard deviation, of which the majority experienced an increase. Approximately 40% of respondents experienced a change in area deprivation between waves (equally split between increased and decreased deprivation), showing substantial residential mobility in this population. A similar pattern of change was observed for individual deprivation.

**Table 2:** **Distribution and transitions of social and economic exposure variables for all young adult respondents (N = 1,260; percentages in parentheses for non-missing respondents and data)**

Exposure variable/transition ^a,b^	Wave 3	Wave 3 to 5 transition	Wave 5	Wave 5 to 7 transition	Wave 7
**Living arrangement**					
Family household (in child role)	620 (52.2%)		440 (38.1%)		350 (30.6%)
Family household (in parent role)	125 (10.6%)		190 (16.5%)		275 (24.0%)
Couple only or with others	190 (16.1%)		250 (21.6%)		285 (24.9%)
One person household	120 (10.2%)		160 (13.9%)		140 (12.2%)
Other multiperson household	125 (10.6%)		115 (10.0%)		95 (8.3%)
Change in living arrangement		370 (34.1%)		365 (34.4%)	
No change in living arrangement		715 (65.9%)		695 (65.6%)	
**Education/Employment**					
In education - not employed	235 (18.7%)		135 (10.7%)		115 (9.1%)
In education - employed	375 (29.8%)		315 (24.9%)		245 (19.4%)
Not in education – not employed (NEET)	145 (11.5%)		145 (11.5%)		155 (12.3%)
Not in education-employed	505 (40.1%)		670 (53.0%)		750 (59.3%)
Change in education/employment		595 (47.2%)		530 (41.9%)	
No change in education/employment		665 (52.8%)		735 (58.1%)	
**Log personal income - CPI adjusted**					
Mean (s.d.)	9.26 (1.15)		9.72 (0.96)		9.98 (0.89)
Increase in personal income by > 0.5 s.d .		460 (36.5%)		330 (26.1%)	
No change		670 (53.2%)		785 (62.1%)	
Decrease in personal income by 0.5 s.d.		130 (10.3%)		150 (11.7%)	
**Area deprivation**					
Quintile 1 (least deprived)	230 (18.8%)		200 (16.5%)		210 (17.2%)
Quintile 2	215 (17.6%)		230 (18.9%)		225 (18.4%)
Quintile 3	245 (20.0%)		265 (21.8%)		250 (20.5%)
Quintile 4	295 (24.1%)		255 (21.0%)		290 (23.8%)
Quintile 5 (most deprived)	240 (19.6%)		265 (21.8%)		245 (20.1%)
Increase in area deprivation		240 (20.3%)		235 (20.0%)	
No change		700 (59.3%)		710 (60.4%)	
Decrease in area deprivation		240 (20.3%)		230 (19.6%)	
**Individual deprivation**					
Nil deprivation factors (least deprived)	750 (61.0%)		735 (60.0%)		665 (54.7%)
1 deprivation factor	240 (19.5%)		275 (22.5%)		270 (22.2%)
2 deprivation factors	115 (9.4%)		110 (9.0%)		150 (12.3%)
3-4 deprivation factors	95 (7.7%)		80 (6.5%)		100 (8.2%)
5 or more deprivation factors (most deprived)	30 (2.4%)		25 (2.0%)		30 (2.5%)
Increase in individual deprivation		255 (21.8%)		195 (18.6%)	
No change		665 (56.8%)		670 (63.8%)	
Decrease in individual deprivation		250 (1.4%)		185 (7.6%)	

^a^Note that all counts are random rounded to a near multiple of 5 as per Statistics New Zealand requirements; therefore numbers in each column do not sum to N = 1,260.

^b^Non-respondents or missings on each variable are not shown.

### Average weekly alcohol consumption

Table 3 shows the results for the linear fixed effects models with average weekly alcohol consumption as the outcome variable.

**Table 3:** **Fixed effects linear regression model of living arrangement, education/employment, income, and deprivation as exposure variables and average weekly units of alcohol consumption as outcome variable**

Model	1: Crude Models ^b^		2: Full model ^c^	
Variable	β	95% CI	β	95% CI
**Living arrangement**				
Other multiperson household	0.61	-0.72,1.93	0.35	-1.00,1.69
One person household	2.51	1.23,3.79	2.32	1.02,3.63
Couple only or with others	-1.23	-2.36,-0.10	-1.37	-2.52,-0.21
Family household (in parent role)	-3.56	-5.13,-1.99	-3.84	-5.44,-2.23
Family household (in child role) (ref.)	0		0	
*p-value* ^a^		*< 0.0001*		*< 0.0001*
**Education/Employment**				
Not in education - employed	0.21	-0.89,1.31	-0.11	-1.36,1.14
Not in education – not employed (NEET)	0.30	-1.10,1.70	0.32	-1.10,1.74
In education - employed	0.16	-0.95,1.28	-0.02	-1.25,1.20
In education -not employed (ref.)	0			
*p-value* ^a^		*0.9772*		*0.9303*
**Increase in log personal income**	0.36	-0.02,0.74	0.18	-0.27,0.62
*p-value* ^a^		*0.0647*		*0.4352*
**One decile increase in area deprivation**	0.01	-0.14,0.16	0.02	-0.14,0.18
*p-value* ^a^		*0.9356*		*0.7747*
**One unit increase in individual deprivation**	0.50	0.14,0.86	0.48	0.10,0.86
*p-value* ^a^		*0.0069*		*0.0132*

^a^Type III Wald tests, which for multichotomous categorical variables (e.g. labour force status) provides a statistical test of the whole construct (not just one non-referent compared to referent comparison).

^b^The crude models include each of the independent variables individually.

^a^The full model includes all independent variables.

We ran separate crude models for changes in each of the social and economic exposure variables (living arrangement, employment/education status, personal income, area deprivation, and individual deprivation) on changes in average weekly alcohol consumption. There was a significant association between changes in living arrangement and individual deprivation with changes in average weekly alcohol consumption. No statistical effect of changes in the other exposure variables on average weekly alcohol consumption was found in the crude models. The impact of changes in living arrangement, and individual deprivation remained significant in the fully adjusted model. The transition over time from living in a family household (in a child role) to living in a one person household was associated with an increase in the average weekly units of alcohol consumption (β = 2.32, 95% CI: 1.02, 3.63), whereas moving in with a partner or spouse was associated with a decrease in units of alcohol consumption (β = -1.37, 95% CI: -2.52, -0.21). In addition, moving into a family household in a parent role (i.e. becoming parent) was associated with a large reduction in units of alcohol consumption per week (β = -3.84, 95% CI: -5.44, -2.23). Experiencing an increase of one unit in self-reported individual deprivation was associated with a small, but significant increase in alcohol consumption (β = 0.48, 95% CI: 0.10, 0.86).

### Frequency of hazardous drinking

Table 4 shows the results for the linear fixed effects models with frequency of hazardous drinking per month as the outcome variable.

**Table 4:** **Fixed effects linear regression model of living arrangement, education/employment, income, and deprivation as exposure variables and frequency of hazardous drinking episodes per month as outcome variable**

Model	1: Crude models ^b^		2: Full model ^c^	
Variable	β	95% CI	β	95% CI
**Living arrangement**				
Other multiperson household	0.49	0.00,0.97	0.29	-0.20,0.79
One person household	0.50	0.04,0.97	0.33	-0.14,0.81
Couple only or with others	-0.28	-0.70,0.13	-0.42	-0.85,0.00
Family household (in parent role)	-0.99	-1.58,-0.41	-1.17	-1.76,-0.57
Family household (in child role) (ref.)	0		0	
*p-value* ^a^		*< 0.0001*		*< 0.0001*
**Education/Employment**				
Not in education - employed	-0.01	-0.43,0.41	-0.18	-0.65,0.30
Not in education – not employed (NEET)	0.09	-0.44,0.63	-0.09	-0.63,0.45
In education - employed	-0.08	-0.50,0.34	-0.20	-0.66,0.27
In education -not employed (ref.)	0		0	
*p-value* ^a^		*0.9066*		*0.8668*
**Increase in log personal income**	0.12	-0.03,0.26	0.12	-0.05,0.29
*p-value* ^a^		*0.1064*		*0.1512*
**One decile increase in area deprivation**	0.06	0.01,0.11	0.06	0.00,0.12
*p-value* ^a^		*0.0277*		*0.0513*
**One unit increase in individual deprivation**	0.20	0.07,0.33	0.25	0.11,0.39
*p-value* ^a^		*0.0030*		*0.0005*

^a^Type III Wald tests, which for multichotomous categorical variables (e.g. labour force status) provides a statistical test of the whole construct (not just one non-referent compared to referent comparison).

^b^The crude models include each of the independent variables individually.

^c^The full model includes all independent variables.

Changes in living arrangement, and both individual and area deprivation were significantly associated with changes in the frequency of hazardous drinking in the crude models. In the full model, the effect size for area deprivation was unchanged (0.06), but with a slight widening of the confidence interval to include the null. As shown for the changes in average weekly alcohol consumption models, if a respondent moved into a family household in the parent role (i.e. becoming a parent), the frequency of hazardous drinking decreased significantly by more than one hazardous drinking session per month (β = -1.17, 95% CI: -1.76, -0.57). Moving into a couple household was also associated with a decrease in the frequency of hazardous drinking episodes per month (β = -0.42, 95% CI: -0.85, 0.00). We also found a 0.25 increase in the monthly frequency of hazardous drinking for young adults who experienced an increase in self-reported individual deprivation by one unit (β = 0.25, 95% CI: 0.11, 0.39).

## Discussion

In this paper, we examined the influence of changes in a range of social and economic factors (modelled both separately and all together) on changes in average weekly alcohol consumption and frequency of hazardous drinking sessions in young adulthood while adjusting for unmeasured confounding through fixed effect regression methods. To date, most studies on drinking behavior in adolescents or young adults have assessed the associations cross-sectionally [12, 28]. Other studies have examined how social and economic factors in childhood or adolescence predict membership of various drinking trajectories (e.g. ‘heavy’ or ‘increasing’) or the development of hazardous alcohol use in young adulthood or later adult life [9, 11, 29–32]. However, none of these studies have controlled for unobserved time-invariant confounding and may therefore have under- or overestimated associations between social and economic measures and drinking behavior.

An original contribution of the present study, compared to previous literature, was separating out the living arrangements of individuals not living at home into living with unmarried partner, with other flatmates or living on their own. Consistent with expectation, the transition from living in a family household (in a child role) into living in a one person household was associated with an increase in the average weekly consumption of alcohol, but no association with frequency of hazardous drinking was found. On the contrary, we found patterns of decreased alcohol consumption and reduced frequency of hazardous drinking for young adults who moved in with their spouse or defacto partner. The latter finding has previously been reported in other studies [17–19].

Moving into a family household in the parent role (i.e. becoming a parent) was associated with a decreased pattern of alcohol consumption and frequency of hazardous drinking sessions. Other studies have also reported that moving into parenthood is associated with decreased alcohol use in larger adult cohorts with a wider age range [20, 33]. Our study, however, shows that this finding is similar for those who become parents in the early years of their adulthood suggesting that this protective effect of moving into more responsible adult roles on drinking behavior may be independent of age. This finding is, however, in contrast to recent research that has shown that those who became parents during their adolescent years showed an increase in their alcohol consumption [22]. This is perhaps not surprising given that other factors associated with drinking behaviors such as finishing education, having a secure income or somewhere to live may also be pertinent issues for those who become teenage parents.

The only economic factor that was associated with changes in drinking behaviors was the self-reported measure of individual deprivation, where increases in deprivation were significantly associated with increases in weekly alcohol consumption and frequency of hazardous drinking. This subjective measure of individual deprivation potentially measures a wider spectrum of both social and economic well-being as it is comprised out of measures such as being able to afford fruit and vegetables, using a food bank, or the need to borrow money for day-to-day needs [25] and has been shown to be strongly associated with changes in mental health [34].

A major strength of this study was repeatedly assessing the same cohort of young adults over time, where all time-invariant confounders (unmeasured heterogeneity), such as early childhood exposures, intelligence, and personality are controlled for in fixed effects regression analysis [16]. However, residual time-varying confounding is possible, such as other major life-changing events (e.g. losing a beloved one), or changes in parental or peer drinking behaviors. Furthermore, the mean-centred estimate that is produced by the fixed-effects linear regression method may be sensitive to health selection (eg, the reversed pathway from drinking behaviors to social and economic outcomes), which, consequently, might have biased the estimates that were found in the present study. However, changes in the social and economic exposure variables were measured over the 12 months prior to the interview date, whereas the drinking behavior variables were measured in the four weeks prior. We therefore argue that there is limited possibility of reverse causation in the model. Although, there is some evidence of an association between engagement in hazardous drinking during adolescence with adverse social and economic outcomes in later adult life [35], there is limited evidence on the reverse pathway within the years of young adulthood. Future research could use structural equation modelling to examine the joint relationship (both directions) between drinking and social and economic factors in young adulthood.

The original SoFIE study population was a nationally representative sample of New Zealand households. The health module which included the questions on drinking behavior, however, was only collected in waves 3, 5 and 7. We have previously shown that younger people of lower socioeconomic status or Māori or Pacific ethnicity were more likely to drop out of SoFIE [23]. Hence, this might have led to selection bias in our study, and led to reduced generalizability of our findings. However, unless these dropout rates were jointly distributed by the social and economic exposure measures and drinking behavior outcome measures, the effect of attrition on the studied associations is likely to be minimal. We have also previously shown that for the association of employment and education with self-rated health there appears to be no difference in the association among those respondents leaving the SoFIE study (non-participants) compared to those respondents who stayed in the study (participants) [36].

It is important to recognize that our findings rely on some of the underlying constructs of fixed-effects regression modelling. Only information on young adults who experienced change in their exposure variables between waves has contributed to the estimates of short-term change in both average weekly alcohol consumption and frequency of hazardous drinking. We can, therefore, not draw any conclusions on young adults who continuously live in a one person or couple household, or who are in the parent role for a longer period of time or live in circumstances of high individual deprivation continuously. Nor can we draw any conclusions on whether the short-term increases or decreases in (observed) average alcohol consumption or frequency in hazardous drinking, lasts over a longer period of time.

## Conclusions

Our findings suggest, and confirm findings of previous studies, that becoming a parent or moving in with a partner or spouse in young adulthood may act as protective factors with regard to alcohol use. Moving into a one person household appears to be associated with at least a short-term increase in the average alcohol consumption of those who move into young adulthood. Moving into more deprivation appears to be associated with an increase in both average weekly alcohol consumption and the frequency of hazardous drinking in young adults. Although more longitudinal research needs to be conducted, a preliminary conclusion from this work is that education on safe drinking should target young people who experience ‘major’ life transitions such as leaving the family home into living alone or moving into more individual deprivation.

## Ethics approval

Ethics approval was obtained for the SoFIE Health module from the University of Otago Ethics Committee.
